# Natural Protein Tolerance and Metabolic Control in Patients with Hereditary Tyrosinaemia Type 1

**DOI:** 10.3390/nu12041148

**Published:** 2020-04-19

**Authors:** Ozlem Yilmaz, Anne Daly, Alex Pinto, Catherine Ashmore, Sharon Evans, Girish Gupte, Saikat Santra, Mary Anne Preece, Patrick Mckiernan, Steve Kitchen, Nurcan Yabanci Ayhan, Anita MacDonald

**Affiliations:** 1 Birmingham Women’s and Children’s Hospital, Birmingham B4 6NH, UK; o.yilmaz@ybu.edu.tr (O.Y.); a.daly3@nhs.net (A.D.); alex.pinto@nhs.net (A.P.); catherine.ashmore@nhs.net (C.A.); evanss21@me.com (S.E.); girishgupte@nhs.net (G.G.); s.santra@nhs.net (S.S.); maryanne.preece@bch.nhs.uk (M.A.P.); steve.kitchen@nhs.net (S.K.); 2 Department of Nutrition and Dietetics, Ankara Yildirim Beyazit University, 06760 Ankara, Turkey; 3 Gastroenterology/ Hepatic/Nutrition, UPMC, Children’s Hospital of Pittsburg, Pittsburg, PA 15224, USA; patrick.mckiernan@chp.ed; 4 Department of Nutrition and Dietetics, Ankara University, 06290 Ankara, Turkey; nyabanci@gmail.com

**Keywords:** tyrosinaemia, natural protein tolerance, metabolic control

## Abstract

In a longitudinal retrospective study, we aimed to assess natural protein (NP) tolerance and metabolic control in a cohort of 20 Hereditary Tyrosinaemia type I (HTI) patients. Their median age was 12 years ([3.2–17.7 years], *n* = 11 female, *n* = 8 Caucasian, *n* = 8 Asian origin, *n* = 2 Arabic and *n* = 2 Indian). All were on nitisinone (NTBC) with a median dose of 0.7 g/kg/day (range 0.4–1.5 g/kg/day) and were prescribed a tyrosine (Tyr)/phenylalanine (Phe)-restricted diet supplemented with Tyr/Phe-free L-amino acids. Data were collected on clinical signs at presentation, medical history, annual dietary prescriptions, and blood Phe and Tyr levels from diagnosis until transition to the adult service (aged 16–18 years) or liver transplantation (if it preceded transition). The median age of diagnosis was 2 months (range: 0 to 24 months), with *n* = 1 diagnosed by newborn screening, *n* = 3 following phenylketonuria (PKU) screening and *n* = 7 by sibling screening. Five patients were transplanted (median age 6.3 years), and one died due to liver cancer. The median follow-up was 10 years (3–16 years), and daily prescribed NP intake increased from a median of 5 to 24 g/day. Lifetime median blood Tyr (370 µmol/L, range 280–420 µmol/L) and Phe (50 µmol/L, 45–70 µmol/L) were maintained within the target recommended ranges. This cohort of HTI patients were able to increase the daily NP intake with age while maintaining good metabolic control. Extra NP may improve lifelong adherence to the diet.

## 1. Introduction

Hereditary Tyrosinaemia type I (HTI) is a rare inherited autosomal recessive disorder caused by reduced activity of fumarylacetoacetate hydrolase (FAH) [1], an enzyme mainly expressed in the liver and kidney. FAH is involved in the last step of tyrosine (Tyr) breakdown [2]. Its reduced activity leads to an accumulation of the toxic metabolites fumarylacetoacetate, maleylacetoacetate and their derivatives succinylacetoacetate [1,3] and increases the risk of developing hepatocellular carcinoma.

The incidence of HTI in the UK is around 1 in 100,000 live births [4]. Many countries conduct newborn screening for HTI due to the availability of an effective treatment option after early diagnosis. Although newborn screening is highly sensitive and specific when using succinylacetone as a primary biochemical marker [5,6,7], HTI is not included in the UK newborn screening programme [4]. Clinical signs of HTI in the neonate include liver failure and renal dysfunction leading to hypophosphatemic rickets [8,9], but the condition may present at a later age [8]. Untreated patients with HTI are at high risk of developing cirrhosis or hepatocellular carcinoma [5,9]. In the early 1990s, nitisinone (2-[2-nitro-4-trifluoromethylbenzoyl]-1,3-cyclohexanedione, NTBC) was introduced, transforming the natural history of HTI. Liver failure was controlled in 90% of patients, chronic liver disease improved, and hepatic manifestations were eradicated provided drug treatment started in infancy [5]. NTBC inhibits 4-hydroxyphenylpyruvate dioxygenase (HDDP), suppressing the production of succinylacetone and related neurotoxic and hepatotoxic metabolites. However, inhibition of the Tyr pathway by NTBC increases Tyr concentrations [10], which can produce ophthalmologic and dermatological complications (keratitis and keratosis, respectively). Therefore, dietary restriction of phenylalanine (Phe) and Tyr is necessary [5].

Although there is a lack of clear consensus, recent management recommendations suggest that blood Tyr concentrations should be maintained between 200 and 400 µmol/L [8,9] and blood Phe concentrations should be kept within the normal reference range [5,9]. Some consider that the minimum blood Phe concentrations should be ≥40 µmol/L, while others suggest they should be ≥50 µmol/L [11]. In order to maintain blood Tyr and Phe within safe target ranges, dietary restriction of both Tyr and Phe is necessary. In practice, this is implemented by restricting natural protein (NP) intake and providing Tyr/Phe-free or low-Tyr/Phe protein substitutes to meet protein requirements [10].

NP tolerance is the maximum daily amount of NP that is tolerated and maintains blood Tyr concentrations within the recommended target range. NP tolerance has been inadequately described in HTI patients on NTBC treatment [10]. Some dietitians suggest that tolerance remains unchanged throughout life [8]. Recently, it has been suggested that NP tolerance is 2–6 g/day in children <2 years old, 5–10 g/day in children aged 2–9 years, 9–10 g/day in adolescents aged 10–14 years and 11–25 g/day in patients aged over 15 years, but the rationale for this recommendation is unclear [10]. Over-restriction of NP intake causes inadequate weight gain, faltering growth and poor wound healing in young children [8,12,13].

In HTI, Phe is restricted because approximately 75% of dietary Phe is hydroxylated to form Tyr [6]. However, very low (below the lower reference range) blood Phe commonly occurs [8,9,10], requiring specific supplementation [8], which may increase blood Tyr [4]. Long-term low blood Phe concentrations are associated with poor growth, neurological deficits and behavioural problems [5,8,13,14,15].

In HTI, it is important to characterise how NP tolerance and metabolic control change over time in order to understand the severity of protein restriction necessary. In this audit, we aimed to collect retrospective, longitudinal data of HTI patients on NTBC treatment following diagnosis.

## 2. Materials and Methods

### 2.1. Project Design

This was a single-centre, longitudinal retrospective case record review of patients with HTI, cared for by Birmingham Women’s and Children’s Hospital. This review only included patients treated with NTBC and a low-protein diet from diagnosis until transition to the adult hospital team (between 16 to 18 years of age) or liver transplantation if this was performed prior to the age of 16 years. Data were collected from medical and dietetic records from 1992 until 2019.

### 2.2. Data Collection

Data were collected on clinical signs at presentation, age at diagnosis, diagnosis/genetics confirmation, parental consanguinity, ethnic origin, family history (e.g., siblings with HTI), medical history, age of starting NTBC, daily dose and frequency of NTBC treatment, other medications, medical and nutritional complications.

Throughout paediatric follow-up, we also collected annual dietary prescriptions: annual median daily NP and total protein intake, protein equivalent amounts from protein substitute intake, vitamin, mineral and other dietary supplementation, and any nutritional support (tube feeding, e.g., nasogastric or gastrostomy) with indications for use. All blood Phe and Tyr levels were reported.

### 2.3. Dietary Assessment

Dietary intake was determined by a 24-h recall from data collected in routine clinics at the time of the final assessment for each patient. Energy and macronutrient intakes were analysed using the Nutritics^®^ software [16]. Nutritional analysis included intake from protein substitutes and special low-protein foods, e.g., low-protein bread or milk.

### 2.4. Dietary Management

All patients were prescribed a low-Tyr/Phe diet characterised by:

(i) Avoidance of high-protein foods including meat, fish, eggs, cheese, nuts and seeds.
(ii) NP allowance (using 1 g protein exchanges), with the amount titrated with blood Tyr concentrations.
(iii) Tyr/Phe-free L-amino acid supplements (protein substitutes) with added vitamins and minerals. Low-Tyr/low-Phe glycomacropeptide-based protein substitutes were not prescribed.
(iv) Provision of low-protein foods, e.g., fruits, some vegetables, oils, sugar and special low-protein foods.
(v) Phe supplements. These were given only if two consecutive Phe levels were below the reference value.

### 2.5. Blood Tyr and Phe Levels

In all patients, we aimed to maintain blood Tyr levels between 200 and 400 µmol/L and blood Phe levels >30 µmol/L. If two blood Phe levels were lower than the reference value (<30 µmol/L), additional Phe was added as a supplement, with a starting dose of 50 mg/day.

### 2.6. Ethical Statement

This review was registered as an audit (CARMS 30467) based on guidelines from the National Research Ethics Service and it did not require ethical review by an NHS Research Ethics Committee.

This was conducted in accordance with basic ethical principles, with respect for participant confidentiality and in line with the Data Protection Act 2000. Written informed consent was obtained from parents/caregivers, and assent from children/adolescents.

### 2.7. Statistics

Data were analysed using descriptive statistics only (percentages, medians, range and means).

## 3. Results

### 3.1. Patients

The demographic information and clinical characteristics of HTI patients are presented in Table 1. Twenty patients (8 Caucasian, 8 Asian, 2 Arabic, 2 Indian origin) diagnosed with HTI were eligible. Fifty-five percent (*n* = 11/20) were females. Ten of 20 patients (50%) were from consanguineous marriages. The median age of patients at the final assessment was 12 years (range 3.2–17.7 years). Five patients required liver transplantation, and one patient died at 3 years of age due to hepatic carcinoma. Three patients (15%) developed behavioural and/or psychological problems, and 10 (50%) had poor school progress, needing extra support.

The median age at presentation was 2 months (range: newborn to 24 months). Four patients (20%) were diagnosed by newborn screening. Of these, three were detected incidentally and followed up due to raised Tyr concentrations on routine newborn screening for phenylketonuria (PKU), and one child was born in a country with universal newborn screening for HTI. Seven (35%) children were siblings of affected cases and underwent early testing. Ten patients (50%) were pre-symptomatic at diagnosis, and five presented with acute liver failure.

### 3.2. Median Prescribed Protein Equivalent from Total Protein, NP and Protein Substitute

Table 2 describes the median prescribed amounts of total protein, NP and protein substitute. 

The median total protein intake (including NP and protein equivalent from protein substitutes) increased with age from 15 g/day at 0–1 year to 84 g/day at 16 years of age. When expressed as g/kg/day, total protein intake decreased from 2.4 g/kg/day at 0–1 year to 1.7 g/kg/day at 16 years of age.

NP intake remained consistent at a median of 5 g/day (range 4–6 g/day) in the first 4 years of life and then gradually increased by 1 g/day each year until the age of 10 years. The median NP intake remained stable at 17 g/day between 10 and 14 years of age and increased to 19 g/day at 15 years and to 24 g/day at 16 years (Table 2).

The median protein equivalent intake from the protein substitute remained consistently above 2 g/kg/day (range 2.1–2.9 g/kg/day) from the first year until the age of 6 years and then gradually decreased to 1.2 g/kg/day by 16 years of age. The protein equivalent from protein substitute provided a median of 79% (range 70% to 88%) of total protein intake between 0 and 16 years of age.

### 3.3. Blood Phe and Tyr Concentrations

**Figure 1:**
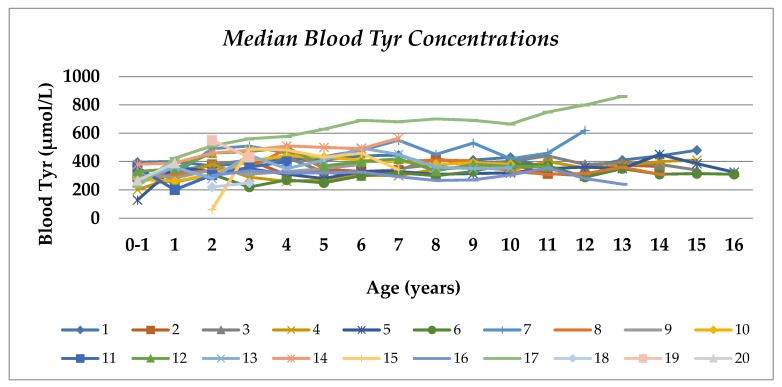
Median blood Tyr concentrations by age in patients with HTI. Coloured lines represent individual patients.

**Figure 2:**
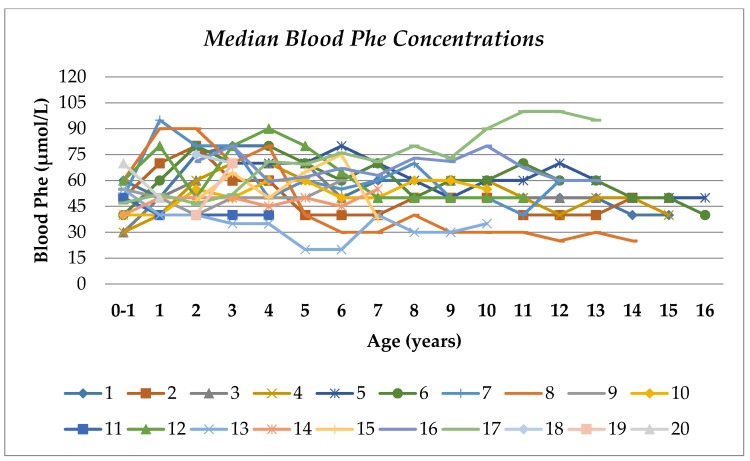
Median blood Phe concentrations by age in patients with HTI. Coloured lines represent individual patients.

Median blood Tyr and Phe concentrations were within the treatment target range (Tyr, 370 µmol/L, range 280–420 µmol/L, and Phe, 50 µmol/L, range 45–70 µmol/L) between 0 and 16 years of age (Table 3, Figure 1 and Figure 2). A median of 66% of all blood Tyr concentrations were maintained within the target range of 200–400 µmol/L, and more than half the subjects (67%) maintained blood Tyr levels within the target range from 5 to 16 years of age (Table 3 and Figure 1). No blood Tyr concentrations were below the target recommendations after the first year of life. The median percentages of blood Tyr concentrations >400 µmol/L and >500 µmol/L were 33% and 7%, respectively.

The median percentage of all blood Phe concentrations ≤30 µmol/L was only 1%. The median percentages of blood Phe concentrations ≤50 µmol/L and ≤100 µmol/L were 55% and 100%, respectively.

### 3.4. Consistency of Blood Tyr and Phe Levels Over Time Compared with NP Intake Changes

**Figure 3:**
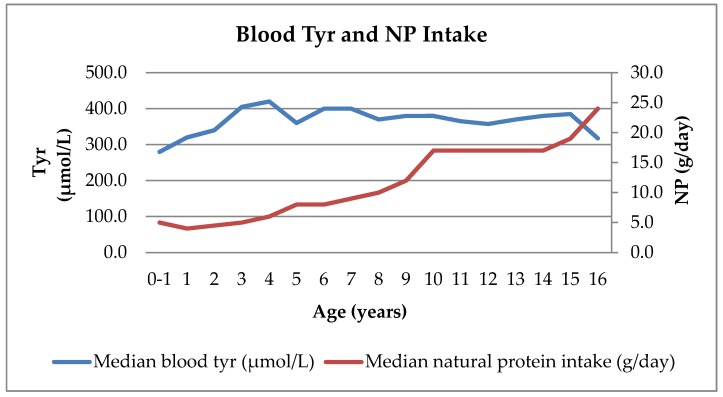
Median blood Tyr concentrations and NP intake by age in patients with HTI.

**Figure 4:**
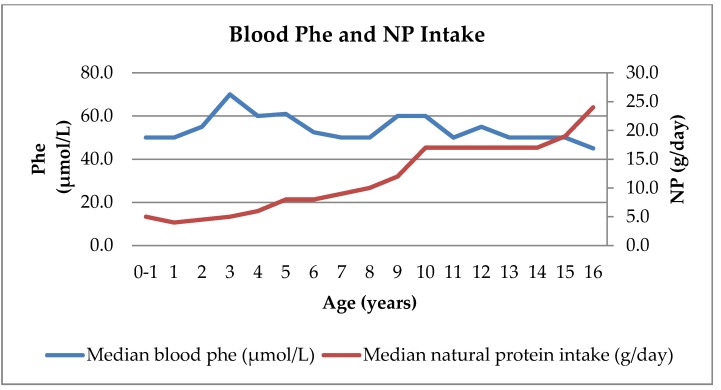
Median blood Phe concentrations and NP intake by age in patients with HTI.

The median blood Tyr concentrations were consistent over time for each age group up to 16 years of age (Figure 3). The median blood Phe concentrations were also stable over time, with a median of 50 µmol/L (range 45–70 µmol/L) (Figure 4). NP intake increased from 5 g/day to 24 g/day while maintaining good metabolic control (Figure 3 and Figure 4).

### 3.5. Actual Dietary Intake from 24 h Recalls 

The 24 h dietary recall analysis at the final assessment (median patient age, 12.4 years), showed that the median energy intake as a percentage of the estimated average energy requirements (EAR) [17] was 92% (range 66%–121%), with 16% (range 7%–22%) of energy from protein, 54% (range 46%–67%) from carbohydrate and 30% (range 16%–41%) from fat (Table 4).

From the 24 h dietary recalls, the actual median intakes (g/day) of: total protein were 77 g/day (range 15–104 g/day); NP 27 g/day (range 8–44 g/day); and protein equivalent from protein substitutes 47 g/day (7–60 g/day).

The median NP intake was 1.1 g/kg/day (range 0.6–1.6 g/kg/day) in children <12 years (*n* = 8) and 0.5 g/kg/day (range 0.4–0.8 g/kg/day) in children >12 years of age (*n* = 8). The median total protein intake was 2.7 g/kg/day (range 1.4–4.2 g/kg/day) in patients <12 years of age (*n* = 8) and 1.6 g/kg/day (range 1.3–2.1 g/kg/day) in patients >12 years of age (*n* = 8).

### 3.6. NTBC

The median NTBC dose administered to patients was 0.7 g/kg/day (range 0.4–1.5 g/kg/day). The majority of patients (*n* = 18, 90%) had one daily dose. Ninety per cent of patients (*n* = 18/20) were prescribed NTBC when diagnosis was established. Median blood NTBC concentrations were 46 μM (15–120 μM) with recommended target ranges varying from 30 to 50 μM [3].

Following treatment with NTBC, plasma succinylacetone was within the reference ranges, except in one child who developed liver carcinoma.

### 3.7. Nutritional Support

Two patients needed nasogastric tube feeding for 3 to 5 years, respectively. Another patient received overnight nasogastric feeding after diagnosis for a limited duration before liver transplantation.

### 3.8. Vitamin and Mineral Supplementation

Three patients were prescribed alfacalcidol, two fat soluble vitamins, and a vitamin and mineral supplement during the review period.

### 3.9. Phe Supplements

Ten patients (50%) received temporary Phe supplementation, usually in the first 2 years of life, at a median dose of 100 mg/day (range 50–250 mg/day) during the review period.

## 4. Discussion

To our knowledge, this is the first longitudinal review describing lifelong changes in NP tolerance together with metabolic control in HTI patients treated with diet and NTBC. The main findings show that NP amounts expressed as g/day increased with age, without deterioration of metabolic control. The median blood Tyr and Phe concentrations remained consistent with increasing age and were maintained within the recommended target ranges for each age group. This NP increase was unexpected and is in contrast to what is observed in classical PKU, where it is well documented that NP tolerance appears to remain unchanged, and blood Phe deteriorates with age [18,19,20].

There have been few reports describing NP tolerance in HTI patients. Previous studies in PKU [12,21] reported that patients tolerate more NP than prescribed when carefully challenged. In a recent study, Pinto et al. [12] showed in 40 well-controlled PKU patients, that additional NP was tolerated while maintaining blood Phe within the target ranges. In our study, the prescribed NP intake increased from a median of 5 g/day in children aged <12 months to 24 g/day by the age of 16 years. Over time NP tolerance varied both between and within the same individuals, and it was likely influenced by growth, adequacy of energy intake, protein substitute adherence and health status, particularly in those patients needing liver transplantation [8,22]. The continual re-appraisal of NP tolerance is essential in HTI patients. Failure to reassess the dietary prescription may contribute to poor dietary adherence and sub-optimal growth [8,10].

In HTI, although recommended target ranges for blood Tyr are commonly discussed, long-term Tyr concentrations are rarely reported or described. Tyr concentrations below 400 μmol/L are considered safe, but recommendations vary between metabolic centres, with some advocating an upper limit of 800 µmol/L [9,10]. However, as a result of the metabolic block induced by NTBC, Tyr concentrations are usually maintained at a level above the normal reference ranges but below the levels associated with ocular complications [9]. In a longitudinal study of 46 French patients on NTBC treatment, dietary adherence was poor, with 36% of patients sustaining elevated blood Tyr concentrations higher than 500 µmol/L [23]. In our study group, the median blood Tyr concentrations were 370 µmol/L, with a median percentage of 66% of all blood Tyr concentrations within the target range of 200–400 µmol/L. This is in line with a previous study from our centre, which showed well-controlled blood Tyr concentrations, with 70% of all concentrations within target range [9] including some patients from this study.

There is no clear consensus about the lower reference range for blood Phe concentrations, but it is advised to maintain concentrations ≥40 µmol/L. In HTI patients, low Phe concentrations, associated with severe NP restrictions, have been reported over many years [9,15,24,25,26]. It is established that blood Phe concentrations are low in the afternoon owing to diurnal variation [9,11,26]. In our audit, the median percentage of all blood Phe concentrations ≤30 µmol/L was only 1%, although a median of 32% of all blood Phe concentrations were ≤30 µmol/L in the first year of life. Ten of 20 patients were prescribed Phe supplements at a dose of 50–250 mg/day for a limited duration during the review period. Low Phe concentrations may impair Phe transport due to the competition of large neutral amino acids across the blood–brain barrier and subsequently cause decreased protein and/or neurotransmitter synthesis in the brain [27]. Therefore in HTI, during rapid periods of brain development, low Phe levels may play a role in neurocognitive issues, but this requires further investigation. Recently, van Ginkel et al. [28] showed the effect of NTBC lowering blood Phe concentrations in HTI mice, leading to potentially high brain Tyr concentrations, although the pathophysiology of these findings is not fully understood. In both healthy infants and children with PKU, very low Phe levels have been associated with a low intelligence quotient (IQ) and delayed development [13,14,29].

Tyr concentrations greater than those in the recommended treatment range are a frequent finding in HTI patients treated with NTBC [23]. In HTI, NTBC enables long-term survival, but there are increasing reports of learning and behavioural problems, with an average IQ score 20 points lower than that of unaffected siblings [30]. Hillgartner et al. [25] suggests NTBC is poorly absorbed across the blood–brain barrier, failing to bind brain HDDP, which leads to under treatment of the central nervous system. Myelin is important for the function of the central nervous system and is formed by oligodendrocytes in which the enzyme FAH is expressed. In HTI, FAH has reduced activity, and poor absorption of NTBC across the blood–brain barrier could lead to the formation of succinylacetone and an increase of aminolevulinate (δALA) in the central nervous system. δALA inhibits myelin formation, leading to cognitive, behavioural or learning disabilities. However, this theory would not explain the learning difficulties observed in patients with HTII or HTIII. Hillgartner et al. [31] studied a HTI mouse model and the effects of NTBC on mouse learning, memory and behaviour. Three sets of mice were tested for memory (wild-type, wild-type + NTBC and HTI + NTBC). Over a series of experiments, the HTI mice had significantly slower test results compared to the two other groups. The HTI mice were able to learn and adopt a learning strategy and had no difficulty with long-term memory. However, it took them significantly longer to learn, they made more errors and failed to adapt easily to change. This mice experiment would suggest that NTBC is not directly responsible for the learning difficulties reported in children.

Suggested mechanisms behind these learning difficulties vary and include hypertyrosinaemia and the increased transport of Tyr across the blood–brain barrier, increased central nervous system dopamine, decreased serotonin or possible oxidative damage from δALA and succinylacetone, which modifies nerve transmission. Baron et al. [32] investigated the relationship between attention-deficit hyperactivity disorder (ADHD), blood Tyr concentrations and cognitive function in eight HTI children. They suggest that high Tyr concentrations inhibit Tyr hydroxylase (TH) and tryptophan hydroxylase 2 (TPH2) activity. Decreased activity of TH and TPH2 with elevated Tyr concentrations impairs the synthesis of dopamine, noradrenaline and serotonin in brain tissue, leading to ADHD. We did not collect psychometric data, but 10 of 20 (50%) patients experienced both behavioural and/or psychological difficulties together with poor school progress.

It is interesting to note that in HTIII, which mimics the biochemical block by NTBC, dietary protein restriction can be stopped in early childhood [8]. HTIII is extremely rare, and the common textbook view is that the majority of patients present with neurological symptoms, normal liver and renal function, with no skin or eye abnormalities despite high concentrations of Tyr. In an early review by Ellaway et al. [33], 9 of 12 patients (75%) had intellectual difficulties, while others remained asymptomatic. In our own cohort of HTIII patients (*n* = 7), all stopped dietary treatment, and the last reported median blood Tyr concentration was 180 μmol/L (range 90–400 μmol/L) and median blood Phe concentration was 50 μmol/L (40–70 μmol/L). This raises the question if HTI behaves in a similar way to HTIII, as the biochemical pathway with NTBC treatment is effectively the same. This may explain why Tyr concentrations remained consistent with increasing patient age. Although neurocognitive delay appears as a common feature in all tyrosinaemia types, with growing evidence pointing to central nervous system dysfunction, it may be that the recommended Tyr concentrations need to be re-evaluated. One possible reason to explain lower Tyr concentrations in HTIII is the ability of Tyr to be converted to 4-hydroxyphenylpyruvate and phenolic compounds, which may be excreted. In early childhood, the development of the enzyme tyrosine amino transferase (TAT) may be a rate-limiting factor in this pathway, necessitating dietary intervention.

It is evident from our results that those who were treated early as a result of newborn screening, either PKU newborn or sibling screening, had an improved clinical outcome (*n* = 11). Of the nine remaining patients, three made a successful recovery once treated with NTBC, one died as a result of liver cancer, and the remaining five needed liver transplantation, as NTBC was unsuccessful in reversing liver damage. In line with previous studies [4,6,34], our audit suggests that early diagnosis through newborn screening is essential to prevent devastating outcomes and improve the prognosis of HTI.

This is the first retrospective review providing long-term follow-up data on the dietary protein intake and blood Phe and Tyr levels in HTI patients. It shows that daily NP tolerance is improved over time, giving greater dietary freedom without compromising the metabolic control. However, this was a retrospective single-centre review, and so there are several limitations to the findings. Patients had variable medical histories, with some requiring hepatic transplant, and the duration of the follow-up varied, factors, which may have influenced the outcomes. Dietary assessments were collected by different dietitians and only provided a snapshot of daily intake; therefore, they may not accurately reflect what patients were consuming.

## 5. Conclusions

The findings of this study highlight the importance of a periodic assessment of NP tolerance in HTI patients to ensure adequate intake and good metabolic control and avoid patient overtreatment. Extra NP may improve lifelong adherence to the diet, especially in adulthood. Despite considerable progress in the diagnosis and management of patients with HTI, there is a need to collect systematic data on dietary practices and overall metabolic control in clinics caring for patients with HTI.

## Figures and Tables

**Table 1:** Demographic information and clinical characteristics of HTI patients.

Patient No.	Gender	Age at Final Assessment (Years)	Ethnicity	Consanguinity	Siblings with HTI	Age at Diagnosis	Diagnosed by Newborn Screening of PKU or HTI	Presenting Symptoms at Diagnosis	Clinical Outcome
1	F	15.2	Caucasian	No	No	2 months	Yes	Pre-symptomatic diagnosis	Dyscalculia, learning difficulties, developmental concerns, anxiety, psychological problems
2	M	14.5	Caucasian	No	No	3 months	No	Acute liver failure and hernia	Behavioural difficulties, psychological problems, poor school progress. Nasogastric feeds for 3 years
3	M	15.2	Asian	Yes	No	Newborn	Yes	Pre-symptomatic diagnosis	Poor school progress, small splenic cyst
4	M	15.1	Arabic	Yes	Yes	Newborn	Sibling screen	Pre-symptomatic diagnosis	Psychological problems poor school progress, seizures
5	F	17.7	Caucasian	No	Yes	Newborn	Sibling screen	Pre-symptomatic diagnosis	Normal
6	M	16.9	Asian	Yes	Yes	Newborn	Sibling screen	Severe liver dysfunction	Poor school progress, alopecia
7	M	13.6	Asian	Yes	Extended family	Newborn	Family screen	Pre-symptomatic diagnosis	Poor school progress
8	F	14.7	Asian	Yes	Yes	4 months	No	Acute liver failure and gastrointestinal bleeding	Normal
9	M	8.3	Caucasian	No	No	3 weeks	Yes	Pre-symptomatic diagnosis	Normal
10	F	10.2	Asian	Yes	No	Newborn	Yes	Pre-symptomatic diagnosis	Poor school progress. Nasogastric feeds for 5 years
11	F	4.7	Caucasian	No	No	3 months	No	Infantile seizures and acute liver failure	Poor school progress
12	M	11.1	Indian	Yes	Yes	Newborn	Sibling screen	Pre-symptomatic diagnosis	Normal
13	M	10.3	Asian	Yes	Yes	Newborn	Sibling screen	Pre-symptomatic diagnosis	Poor school progress. Early coagulopathy
14	F	8.3	Asian	Yes	Yes	Newborn	Sibling screen	Pre-symptomatic diagnosis	Poor school progress. Early coagulopathy
15	F	6.3 *	Caucasian	No	No	22 months	No	Rickets, hepatomegaly, glycosuria, myopathy, respiratory muscle weakness	Cirrhosis
16	M	13.3 *	Caucasian	No	No	20 months	No	Cirrhosis and portal hypertension	Hepatocellular carcinoma with progression of tumour
17	F	12.7 *	Indian	No	No	3 months	No	Liver failure, hyperinsulinism, feeding problems, congenital cytomegalovirus infection	Compensated cirrhosis
18	F	3.4 *	Asian	Yes	No	24 months	No	Acute liver failure, vomiting, mild fever	Cardiomyopathy
19	F	3.2 *	Arabic	No	No	3 months	No	Hepatosplenomegaly, abdominal distension	Lymphadenopathy
20	F	Died	Caucasian	No	No	10 months	No	N/A	Died at age of 3 years due to hepatocellular carcinoma

Abbreviations: HTI: Hereditary Tyrosinaemia Type 1; PKU: Phenylketonuria; F: female; M: male. * Age at transplantation.

**Table 2:** The median amounts of total protein, natural protein (NP) and protein equivalent intake from protein substitutes prescribed by age.

		Total Protein				Natural Protein				Protein Equivalent from Protein Substitutes			
		g/day		g/kg/day		g/day		g/kg/day		g/day		g/kg/day	
Age (years)	No. Subjects Each Age Group	Median	IQR	Median	IQR	Median	IQR	Median	IQR	Median	IQR	Median	IQR
0–1	7	15	3	2.4	0.8	5	2	0.8	0.4	11	3	1.5	0.6
1	13	28	9	3.4	0.5	4	2	0.4	0.2	25	9	2.9	0.9
2	19	34	9	2.9	0.4	5	3	0.5	0.3	29	5	2.4	0.3
3	19	38	9	2.8	0.6	5	5	0.4	0.4	31	12	2.4	0.7
4	15	44	8	2.5	0.9	6	3	0.4	0.3	38	10	2.2	0.8
5	15	47	8	2.6	0.6	8	5	0.4	0.4	38	9	2.3	0.4
6	14	50	9	2.4	0.6	8	5	0.4	0.2	42	8	2.1	0.6
7	14	53	8	2.4	0.6	9	7	0.4	0.2	45	5	1.9	0.6
8	14	55	9	2.1	0.4	10	5	0.4	0.2	45	2	1.8	0.3
9	12	57	11	1.9	0.4	12	9	0.4	0.2	45	5	1.6	0.3
10	12	63	13	1.8	0.6	17	9	0.5	0.2	45	5	1.4	0.4
11	10	66	16	1.6	0.6	17	7	0.4	0.2	48	15	1.3	0.3
12	9	76	18	1.4	0.7	17	3	0.4	0.1	60	15	1.1	0.5
13	8	78	11	1.4	0.4	17	3	0.3	0.1	60	0	1.0	0.3
14	7	79	9	1.2	0.5	17	4	0.4	0.1	60	0	1.0	0.3
15	4	86	12	1.6	0.4	19	7	0.4	0.2	60	5	1.1	0.4
16	1	84	0	1.7	0.0	24	0	0.5	0.0	60	0	1.2	0.0

Abbreviations: IQR: interquartile range.

**Table 3:** Median blood Tyr and Phe concentrations by age in patients with HTI.

Age (years)	No. Subjects in Each Age Group	Blood Tyr (µmol/L)		% of Blood Tyr Concentrations										Blood Phe (µmol/L)		% of Blood Phe Concentrations					
				<200 µmol/L		200–400 µmol/L		>400 µmol/L		>500 µmol/L		>600 µmol/L				≤30 µmol/L		≤ 50 µmol/L		≤ 100 µmol/L	
		Median	IQR	Median	IQR	Median	IQR	Median	IQR	Median	IQR	Median	IQR	Median	IQR	Median	IQR	Median	IQR	Median	IQR
0–1	16	280	60	20	16	58	15	12	16	3	6	0	4	50	16	32	23	64	33	98	5
1	16	320	79	12	14	60	16	28	27	7	12	1	7	50	23	20	22	58	37	96	17
2	19	340	113	0	6	66	50	20	51	3	22	0	1	55	32	13	12	50	50	97	10
3	19	405	116	0	2	52	45	46	48	13	37	0	13	70	19	9	11	29	40	97	6
4	17	420	163	0	2	44	52	56	55	21	40	8	14	60	21	6	10	32	42	100	4
5	16	360	107	0	0	62	51	38	57	12	22	0	5	61	20	10	14	34	57	99	5
6	16	400	135	0	1	54	65	46	68	12	31	1	10	53	17	7	9	52	54	100	3
7	15	400	113	0	0	68	51	32	56	6	38	0	13	50	17	0	14	53	56	100	1
8	13	370	75	0	2	67	40	33	41	6	17	0	2	50	10	2	7	55	40	100	0
9	13	380	55	0	0	60	23	40	23	3	14	0	0	60	10	0	2	36	56	100	0
10	13	380	60	0	0	75	22	25	22	8	10	0	3	60	10	0	8	46	44	100	0
11	11	365	70	0	0	66	23	34	27	8	16	0	0	50	19	0	4	72	52	100	0
12	10	358	71	0	0	78	24	22	28	1	6	0	0	55	18	1	8	63	67	100	0
13	9	370	40	0	0	71	18	29	18	2	3	0	0	50	10	0	4	83	51	100	0
14	7	380	83	0	2	67	37	33	45	11	15	0	5	50	5	0	10	82	13	100	0
15	5	385	70	0	0	60	32	40	32	11	20	0	7	50	10	0	0	79	20	100	0
16	2	318	8	0	0	100	0	0	0	0	0	0	0	45	5	0	0	98	3	100	0

Abbreviations: IQR: interquartile range; Tyr: tyrosine; Phe: phenylalanine.

**Table 4:** Dietary intake of patients with HTI based on 24-h food recall at the final assessment.

Patient No.	Gender	Age at Dietary Assessment (years)	Energy (kcal/d) (EAR %)	Protein							Carbohydrate			Fat		
				Total (g/day)	Total (g/kg/day)	Natural (g/day)	Natural (g/kg/day)	PE from PS (g/day)	PE from PS (g/kg/day)	% of Energy	(g/day)	(g/kg/day)	% of Energy	(g/day)	(g/kg/day)	% of Energy
**1**	F	15.2	1871 (78)	83	1.4	23	0.4	60	1.0	18	242	3.8	52	64	1.0	30
**2**	M	14.5	2590 (121)	86	1.7	26	0.5	60	1.2	13	386	7.5	60	78	1.5	27
**3**	M	15.2	2475 (87)	87	1.3	27	0.4	60	0.9	14	286	4.2	46	109	1.6	40
**4**	M	15.1	1776 (96)	91	1.5	30	0.5	60	1.0	21	263	4.3	59	40	0.6	20
**5**	F	17.7	1638 (66)	91	1.8	31	0.6	60	1.2	22	207	4.2	51	50	1.0	27
**6**	M	16.9	2303 (74)	104	1.9	44	0.8	60	1.1	18	322	5.9	56	66	1.2	26
**7**	M	13.6	2252 (93)	95	2.1	35	0.8	60	1.3	17	376	8.3	67	40	1.0	16
**8**	F	14.7	2223 (94)	71	1.4	26	0.5	45	0.9	13	320	6.5	58	71	1.4	29
**9**	M	8.3	1887 (108)	65	2.5	25	1.0	40	1.5	14	256	9.7	54	67	2.5	32
**10**	F	10.2	1957 (101)	79	1.6	30	0.6	49	1.0	16	269	5.7	55	62	1.3	29
**11**	F	4.7	1556 (114)	72	4.2	27	1.6	45	2.6	19	194	11.4	50	54	3.2	31
**12**	M	11.1	1919 (90)	75	2.5	32	1.1	43	1.4	15	286	9.5	60	53	1.8	25
**13**	M	10.3	1703 (83)	71	2.9	26	1.1	45	1.8	17	226	9.2	53	57	2.3	30
**14**	F	8.3	1755 (108)	72	3.4	27	1.3	45	2.1	16	235	11.0	54	58	2.7	30
15 *	F	6.3	Liver transplant													
16 *	M	13.2	Liver transplant													
17 *	F	12.7	Liver transplant													
**18**	F	3.4	961 (89) Liver transplant	41	2.9	11	0.8	30	2.1	17	144	10.1	60	25	1.7	23
19 *	F	3.2	Liver transplant													
**20**	F	Died 3years	906 (84)	15	1.4	8	0.7	7	0.7	7	119	10.8	52	42	3.8	41

Abbreviations: EAR: estimated average requirements; F: female; M: male; PE: protein equivalent; PS: protein substitute * There were no available accurate dietary histories taken prior to transplantation.
